# Conversational AI in Hereditary Cancer Care: Sociotechnical Study of Responsible Design Requirements

**DOI:** 10.2196/97869

**Published:** 2026-09-30

**Authors:** Dominic Lammert, Jacqueline Lammert, Justin Hofenbitzer, Tristan Radtke, Yingping Sun, Martin Haimerl, Stefanie Betz, Doran Holger Scholl, Jürgen Pfeffer

**Affiliations:** 1 Chair of Computational Social Science, TUM School of Social Science and Technology, Technical University of Munich, Hochschule für Politik München, Richard-Wagner-Straße 1, Munich, Bavaria, 80333, Germany, +49 89907793200; 2 Chair of Medical Informatics, Institute for AI and Informatics in Medicine, TUM School of Medicine and Health, TUM University Hospital, Technical University of Munich, Munich, Germany; 3 Chair of Law and Regulation of the Digital Transformation, Department of Governance, TUM School of Social Sciences and Technology, Technical University of Munich, Munich, Germany; 4 School of Business, Shandong Normal University, Jinan, Shandong, China; 5 Faculty II: Engineering and Technology, Furtwangen University, Furtwangen, Baden-Wurttemberg, Germany; 6 Faculty I:, Computer Science and Applications, Furtwangen University, Furtwangen, Baden-Wurttemberg, Germany; 7 School of Engineering Sciences, Software Engineering, Lappeenranta-Lahti University of Technology (LUT), Lappeenranta, Finland

**Keywords:** BRCA1/2, conversational AI, responsible AI, participatory design, genetic counseling, patient decision support, breast cancer gene 1 and 2

## Abstract

**Background:**

Individuals with *BRCA1/2* germline variants face complex, preference-sensitive medical decisions under psychological distress. Structural constraints in genetic counseling create support gaps during waiting periods, prompting patients to rely on fragmented or misleading online information. Conversational AI may offer low-threshold, continuous informational support, yet methods for its responsible integration into clinical care remain unclear.

**Objective:**

This study identifies and systematizes stakeholder-informed sociotechnical design requirements and governance conditions for a clinically bounded conversational AI system intended to support comprehension, appraisal, and appropriate use of complex hereditary cancer information in *BRCA1/2*-related care.

**Methods:**

We conducted an exploratory, qualitative, predevelopment requirements study using 2 structured participatory workshops with interdisciplinary stakeholders (N=18). Guided exercises included stakeholder analysis, the value proposition canvas, and the sustainability awareness framework. Structured workshop outputs, field notes, and artifacts were analyzed using inductive thematic analysis.

**Results:**

Stakeholder workshops revealed that responsible design was shaped less by consensus around standalone responsible AI principles than by three recurrent cross-stakeholder tensions: (1) accessibility and continuity of support versus clinical role boundaries, (2) simplification and emotional reassurance versus medical precision and epistemic transparency, and (3) technical scalability versus governance, data protection, and institutional accountability. These tensions translated into design requirements for constrained system authority, mandatory human oversight, context-sensitive communication, and integration into clinical and regulatory governance structures.

**Conclusions:**

Conversational AI in this context should be explicitly constrained and embedded within clinical governance structures to be perceived as responsible. Responsible implementation requires transparent design, human oversight, integration into existing care pathways, and early alignment with German/European Union (EU) regulatory requirements concerning data protection, medical device classification, genetic data, and AI transparency.

## Introduction

### Overview

Individuals carrying pathogenic germline variants in *BRCA1/2* face complex, preference-sensitive medical decisions regarding risk-reducing surgeries and surveillance, often under significant psychological distress with long-term implications for physical and mental health [1,2]. Despite the importance of genetic counseling, clinical pathways often lack continuous support beyond initial disclosure, while limited consultation time constrains the ability to address informational and psychosocial needs [3].

In Germany and internationally, structural bottlenecks and prolonged waiting times for specialized second opinions create a critical informational gap during the decision-making period. Patients frequently turn to online resources, where fragmented or misleading information may distort risk perception and increase uncertainty [4-6].

Conversational agents, particularly chat and voice bots, are increasingly discussed as low-threshold tools in health care. While advances in large language models (LLMs) have improved accessibility, challenges regarding reliability and ethical governance remain [7,8]. This study does not assume that patients lack access to conversational AI through general-purpose systems. Rather, it asks what added value a purpose-built, institutionally governed, and clinically bounded system could provide in *BRCA1/2*-related care. The envisioned conversational AI system is conceptualized as a supportive tool for waiting periods that complements, rather than replaces, human expertise.

Building on responsible AI principles, this study examines how ethical considerations can be translated into concrete design requirements for sensitive clinical contexts. While prior research has focused on the accuracy and usability of medical chatbots, empirical work on how responsibility is operationalized in emotionally sensitive, preference-sensitive decision contexts remains limited. This study therefore investigates how stakeholder perspectives can inform the responsible design and future integration of conversational AI into hereditary cancer care.

Using a qualitative sociotechnical approach, the study combines stakeholder analysis [9], the value proposition canvas (VPC) [10], and the sustainability awareness framework (SusAF) [11] to examine how digital health tools can be ethically integrated into the organizational and relational context of hereditary cancer care and how they shape responsibility, knowledge mediation, and patient autonomy. This study derives actionable design implications for the responsible integration of conversational AI into clinical care pathways, grounded in interdisciplinary stakeholder perspectives.

As an early-stage, stakeholder-informed design inquiry, this study did not include direct patient participation in the initial phase. Patient involvement is addressed as a methodological limitation and as a requirement for subsequent phases of system development.

This study contributes by translating abstract principles of responsible AI into concrete, stakeholder-informed design requirements and governance conditions for conversational AI in sensitive clinical contexts. In particular, it foregrounds legal and regulatory requirements not as external constraints to be addressed after design but as constitutive design conditions shaping system boundaries, data protection, medical device classification, genetic data governance, AI transparency, and institutional accountability in the German/European Union (EU) context.

### Background

### Clinical Context: *BRCA1/2* Mutation Carriers

Carriers of *BRCA1/2* germline variants face substantially increased cancer risks, requiring complex decisions on prophylactic surgery and surveillance, with implications for psychological well-being, family planning, and quality of life [1,2,12-14].

Standard genetic counseling, often limited to a pretest and disclosure session, frequently fails to meet patients’ longitudinal needs. Psychological distress at the time of result disclosure impairs information processing, while limited consultation time constrains clinicians’ ability to address psychosocial concerns in depth [3].

In Germany, specialized second-opinion services exist through the German Consortium for Familial Breast and Ovarian Cancer (FBREK), yet structural bottlenecks result in waiting times of up to 6 to 8 weeks. This gap contributes to patient overload and delayed decision-making, particularly for non-German-speaking individuals [15-17]. International evidence confirms similar deficits; for example, a recent Facing Our Risk of Cancer Empowered (FORCE) survey (n=1261) reported substantial gaps in posttest support, particularly regarding surgical menopause and family risk communication [4].

This “support vacuum” is often filled by online resources, where fragmented, inconsistent, or misleading information can distort risk perception and increase uncertainty [5,18-20]. In *BRCA1/2*-related hereditary cancer care, these risks are amplified by the complexity of decisions involving probabilistic cancer risk, preventive surgery, surveillance, fertility implications, family communication, and psychosocial consequences. Importantly, access to digital health information does not necessarily imply the ability to interpret, evaluate, or apply that information appropriately. Patients’ perceived confidence in navigating online resources may diverge from their actual health literacy or eHealth literacy, particularly when information is technical, emotionally charged, or clinically preference-sensitive. Consequently, there is a substantial unmet need for accessible, evidence-based support that strengthens comprehension, appraisal, contextualization, and appropriate use of health information in preparation for clinical decision-making.

### Chat and Voice Bots as Support for *BRCA1/2* Mutation Carriers

The evolution of conversational AI began with ELIZA [21] and PARRY [22], early systems rooted in mental health applications. Today, medical chatbots are deployed across domains, though ethical and implementation challenges remain significant [7,8,23]. In oncology, they support screening, symptom tracking, and risk communication [24,25], with reported high user satisfaction, for example, in breast cancer education [26].

This study addresses the previously described “support vacuum” for *BRCA1/2* mutation carriers during waiting periods for clinical consultations. A conversational chat and voice bot is conceptualized as a supportive tool that provides evidence-based, interactive information to assist in preparing for clinical encounters.

At this stage, the chat and voice bot represents a conceptual system design rather than an implemented prototype. The envisioned architecture would combine an LLM with retrieval-augmented generation (RAG) to ground responses in a curated, evidence-based knowledge base. The proposed content development process would follow established standards such as the International Patient Decision Aid Standards (IPDAS) and would build on materials from the FBREK Consortium and the BRCA Network. The system is envisioned to support multilingual use and varying levels of health literacy.

In the proposed design, system integrity would depend on automated cross-referencing with the knowledge base, regular expert review, and clearly defined content governance processes. These mechanisms should be understood as design requirements derived from stakeholder input rather than as implemented or technically validated system features. Accordingly, Figure 1 illustrates the planned system architecture, not a deployed system.

**Figure 1.**
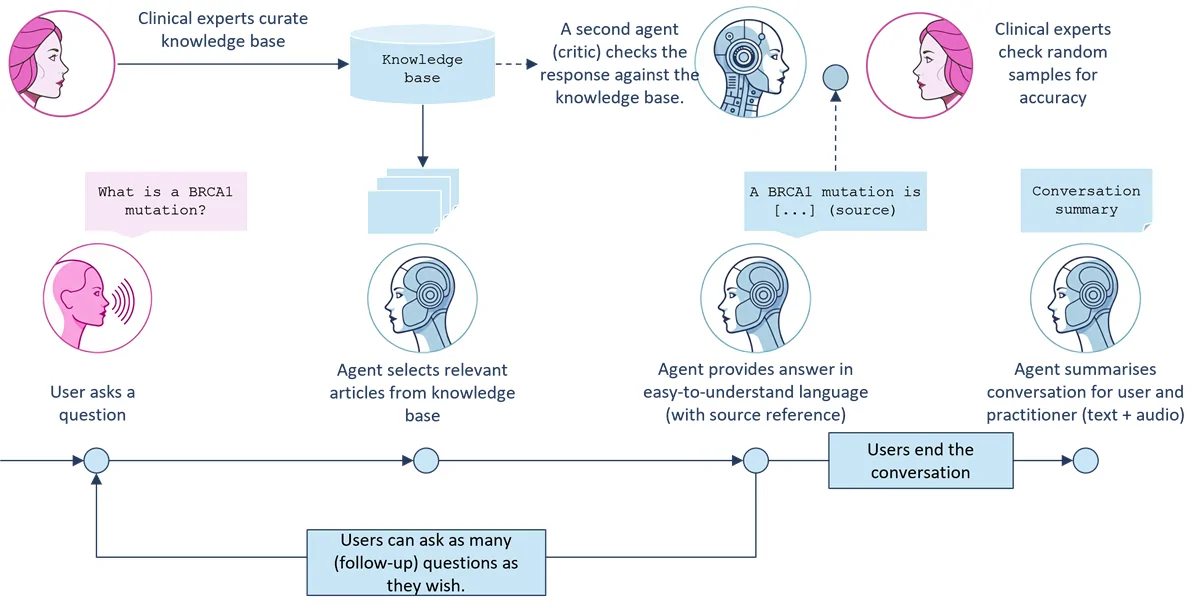
Planned system architecture: a clinical knowledge base integrated via retrieval-augmented generation (RAG) to provide evidence-based support.

The rationale for such a purpose-built system is not that general-purpose LLMs are unavailable or incapable of conversational interaction. Rather, its added value lies in clinical and institutional governance. In contrast to general-purpose LLMs, the envisioned system would be restricted to a clearly defined *BRCA1/2*-related informational and preparatory role, grounded in a curated and expert-reviewed knowledge base, aligned with established patient decision-aid standards, and connected to escalation pathways for human support. The system is therefore conceptualized not as a more autonomous alternative to existing LLMs, but as a clinically bounded sociotechnical arrangement that limits unsupported interpretation, clarifies accountability, and supports preparation for clinical encounters.

A single example illustrates what this constraint adds. A 28-year-old carrier whose mother died of ovarian cancer at 42 years asks whether a positive BRCA1 result means she will certainly develop the disease. An unconstrained general-purpose system typically answers with cumulative lifetime incidence, approximately 44% by age 80 years [1], names risk-reducing salpingo-oophorectomy without temporal context, and closes with a generic instruction to consult a physician. Each element is accurate in isolation, yet together they equate genetic predisposition with imminence for a user in acute distress. A clinically bounded system would instead be configured to set that figure against the age-stratified incidence for her own age band, which remains an order of magnitude lower over the coming years [1]; to locate the surgical discussion in its guideline-anchored window from about 35 years of age for BRCA1 carriers, adjusted to the earliest diagnosis in her family [13,14]; and to route her to the responsible contact in her own care pathway. The difference lies not in model capability or conversational tone, but in the curated knowledge base, the response constraints, and the escalation rules for which the institution remains accountable.

Functionally, the envisioned system would provide structured summaries in text and audio formats to support clinical consultations and would include escalation pathways to human professionals for users in psychological distress.

### From Ethical Principles to Design Practice: Responsible AI

Conversational agents have the potential to improve accessibility and user experience [27-29], but challenges remain regarding inclusivity, reliability, and ethical governance [30-34]. This study adopts a socioinformatics perspective, conceptualizing the system as embedded within social and organizational contexts where design choices shape care practices [35-37]. Responsible AI provides a framework to address these challenges, emphasizing transparency, fairness, and accountability, while highlighting the need to address issues such as explainability and bias [38-43].

In this study, responsible AI is understood as the design, development, and governance of AI systems in ways that are lawful, ethically aligned, technically and socially robust, transparent, accountable, privacy-preserving, and subject to meaningful human oversight. The EU High-Level Expert Group’s Trustworthy AI guidance serves as the primary reference point for operationalizing this understanding, particularly through its emphasis on human agency and oversight, technical robustness and safety, privacy and data governance, transparency, diversity, nondiscrimination and fairness, societal and environmental well-being, and accountability [39].

These principles were used as sensitizing concepts rather than as a deductive coding scheme. Human oversight informed the analysis of role boundaries, escalation pathways, and the positioning of medical professionals as primary decision makers. Transparency and explainability guided attention to uncertainty communication, disclosure of system limitations, and prevention of automation bias. Privacy and data governance informed the analysis of genetic data sensitivity, consent, data minimization, and institutional accountability. Technical robustness and safety informed requirements for curated knowledge bases, expert review, and constrained system authority. Fairness and societal well-being informed attention to multilingual access, health literacy, eHealth literacy, accessibility, and the prevention of exclusion.

To operationalize these principles, the study draws on participatory design and value-sensitive design (VSD) to integrate stakeholder perspectives into system development [9,44,45]. Accordingly, responsible AI is treated not as a fixed checklist but as a sociotechnical design task involving the translation of general principles into concrete governance conditions, communication strategies, and role boundaries for a clinically bounded conversational AI system in *BRCA1/2*-related hereditary cancer care.

### Research Gap and Research Question

Despite advances in digital health, limited evidence exists on how conversational AI can responsibly support *BRCA1/2* mutation carriers during waiting periods between genetic counseling, second opinions, and clinical decision-making. The underexplored issue is not simply whether conversational AI can provide information in genetic medicine, but how such systems can support the comprehension, appraisal, contextualization, and appropriate use of complex hereditary cancer information in emotionally charged and preference-sensitive contexts.

Existing research on medical chatbots has focused primarily on technical accuracy, usability, and user satisfaction, while less attention has been paid to how conversational AI can remain bounded by clinical governance, human oversight, validated content, escalation pathways, and responsible AI principles. In *BRCA1/2*-related care, this is particularly important because users must interpret probabilistic risk information and preventive options without mistaking informational support for clinical decision-making authority.

This study addresses this gap through a stakeholder-informed, sociotechnical perspective. It is guided by the following research question: how can stakeholder-informed design requirements for the responsible design and future integration of a conversational AI system in clinical care pathways be identified?

## Methods

### Overview

This study used a qualitative, practice-oriented design to identify stakeholder-informed design requirements for the responsible development of a conversational AI system in hereditary cancer care. The system served as an empirical case to examine sociotechnical and ethical dynamics. The study focused on deriving context-sensitive design requirements rather than evaluating system performance or user outcomes.

The conversational AI system examined in this study represents a conceptual design case rather than a fully implemented or deployed product. No prototype was implemented, no technical validation was performed, and no content pipeline was operationalized as part of this study.

The study followed a qualitative exploratory design informed by interpretive research principles and is reported in accordance with established standards, including the COREQ (Consolidated Criteria for Reporting Qualitative Research), to enhance transparency and rigor. In addition, relevant items from the CONSORT-EHEALTH (Consolidated Standards of Reporting Trials of Electronic and Mobile Health Applications and Online Telehealth) checklist were considered where applicable to enhance transparency in reporting digital health research.

The methodology integrated 3 complementary frameworks, including stakeholder analysis [9], the VPC [10], and the SusAF [11], which together structure data collection and analysis across sociotechnical dimensions (Figure 2).

**Figure 2.**
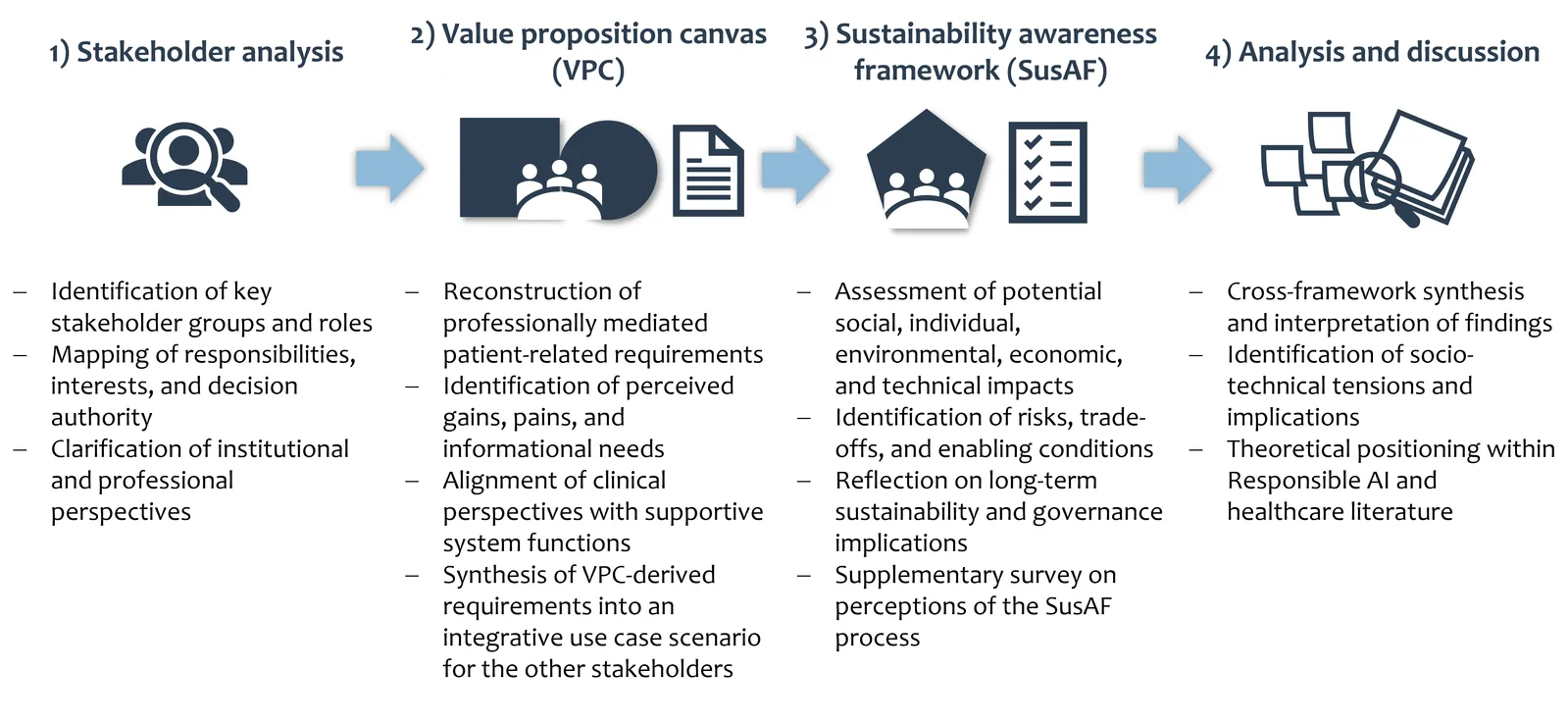
Sequential analytical work steps applied in the study.

### Stakeholder Analysis

The stakeholder analysis followed a 4-step process (identification, relevance assessment, interaction, and solution development) based on established participatory design approaches [9].

An interdisciplinary core team identified relevant stakeholders across clinical, technical, and social domains. For each group, key characteristics, including interests, perceived influence (scale 1‐10), and conflict potential (scale 1‐10), were assessed (Table 1).

**Table 1.** Stakeholder overview (abbreviated) with influence and conflict potential.

Stakeholder^a^	Known interests	Assumed interests	Perceived influence	Conflict potential	Present in the workshop
Patients	Clear, trustworthy info	Emotional relief; control	10	8	No
Oncologists	Preference-sensitive decisions	Time savings; clarity	10	8	Yes
Surgeons	Safe, aesthetic surgery	Manage expectations	9	7	Yes
Radiologists	Structured screening	Clarify false positive risk	9	7	Yes
Nursing staff	Coordination	Reduce cancellations	9	7	Yes
Patient advocacy	Participation; access	Strengthen autonomy	8	8	Yes
Psychologists	Psychosocial continuity	Early crisis detection	8	7	Yes
Ethicists	Ethical reflection	Influence guidelines	8	6	Yes
Hospital IT	IT security	Integration	7	5	Yes
UI^b^/UX^c^ designers	User-centered design	Reduce cognitive load	7	3	Yes
Health insurers	Cost control; quality	Prevent misinformation	7	3	Yes
Disability groups	Accessibility	Avoid digital exclusion	6	4	Yes
Linguists	Cultural sensitivity	Prevent misunderstandings	6	4	Yes
Data protection experts	GDPR^d^ compliance	Legal risk reduction	5	5	Yes
Media educators	Explain complexity	Improve transparency	5	3	Yes

a For each stakeholder group, perceived influence and conflict potential were assessed on a 1-10 scale through consensus-based discussion by the interdisciplinary core team. These scores represent qualitative assessments specific to the investigated use case rather than empirically measured quantities.

b UI:user interface.

c UX: user experience.

d GDPR: General Data Protection Regulation.

To ensure analytical focus, only stakeholders with an influence score of 5 or higher were included in participatory workshops, while additional stakeholders relevant for later implementation phases were documented but not actively involved. This threshold reflects a balance between inclusiveness and feasibility in early-stage participatory design.

While patients represent the primary end users, direct patient involvement was not included in this initial exploratory phase. This was an ethics-oriented precautionary decision within a phased predevelopment process, rather than an empirically demonstrated indication that patient involvement would have been harmful, inappropriate, or infeasible. To incorporate patient-related perspectives at this stage, psycho-oncology experts and patient advocacy organizations with experience in *BRCA1/2*-related care were included as professional stakeholders. Consequently, the findings reflect stakeholder-mediated interpretations of patient needs rather than direct patient accounts. Direct patient involvement is planned as a necessary next step to validate, refine, and potentially challenge the stakeholder-derived requirements once system boundaries, safeguards, and governance conditions have been further specified.

A stakeholder mapping was conducted to visualize relationships and cluster actors across medical, social, technical, financial, and patient-related domains (Figure 3).

**Figure 3.**
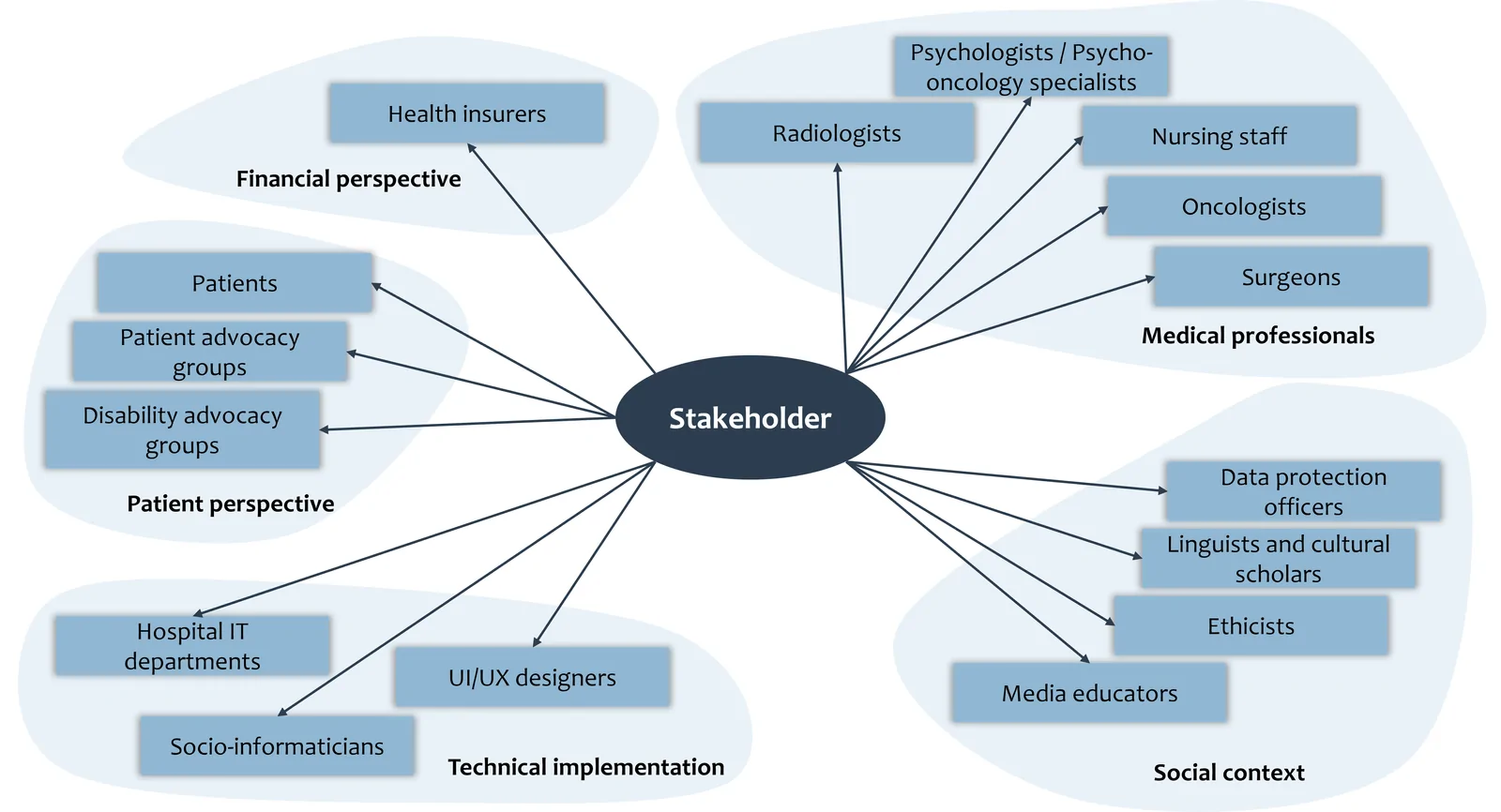
Stakeholder mapping for the medical chat and voice bot.

Participants were recruited using purposive sampling to ensure representation of key stakeholder groups relevant to hereditary cancer care and conversational AI governance, based on professional expertise and practical involvement.

Participatory interactions informed the identification of key tensions, synergies, and unmet needs. Data from workshop outputs, field notes, and artifacts were analyzed iteratively to derive design requirements.

### VPC

The VPC [10] was used as a co-design instrument to align the needs of *BRCA1/2* mutation carriers with clinically appropriate and technically feasible system components (Figure 4). The workshop involved medical professionals from relevant disciplines, complemented by technical and socioinformatics expertise.

**Figure 4.**
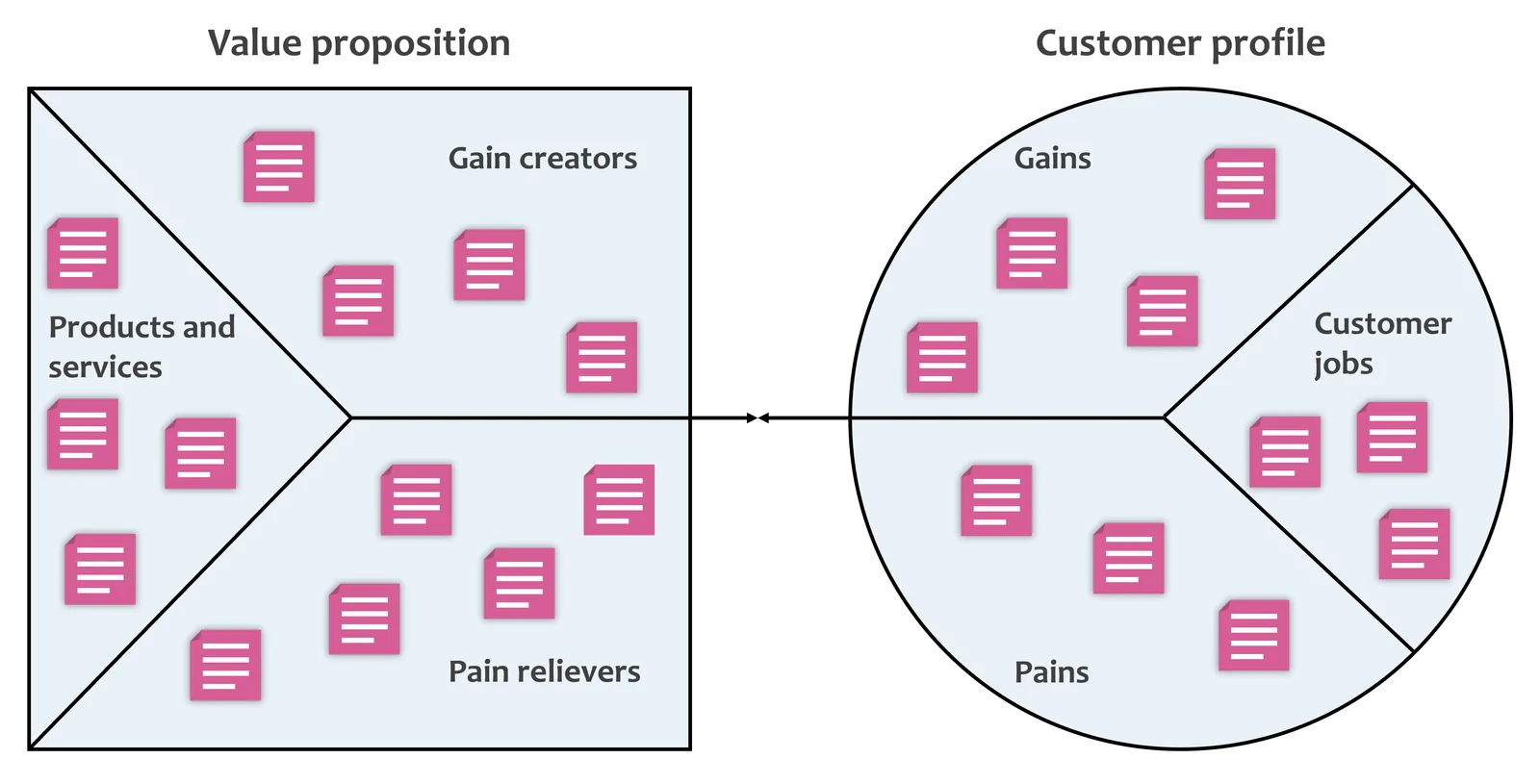
Value proposition canvas (VPC).

Within the customer segment, the workshop characterized informational needs, emotional burdens, and expectations regarding counseling. The value map translated these into system functionalities and communicative boundaries, positioning the chat and voice bot as a supportive tool rather than a decision-making authority.

The workshop resulted in a consolidated one-page user-case scenario, which served as a shared reference for subsequent participatory sessions and supported alignment between clinical requirements and system design (see Multimedia Appendix 1).

### SusAF

The SusAF was applied as a participatory instrument to examine the broader sociotechnical implications of the chat and voice bot. In contrast to the VPC workshop, the SusAF session involved all identified stakeholder groups (n=18) to capture a wide range of perspectives.

The framework structures analysis across 5 sustainability dimensions and 3 orders of effects, enabling the identification of both immediate and long-term impacts [11] (Tables 2 and 3).

Participants identified and discussed potential positive, negative, and ambivalent effects, which were subsequently prioritized using an impact matrix based on likelihood and significance (Figure 5). This process enabled the identification of key factors that informed design requirements.

Results were further structured using the sustainability awareness diagram (SusAD) to map impacts across dimensions and effect levels, supporting interdisciplinary reflection and making implicit risks explicit (Figure 6).

A final stakeholder survey confirmed the usefulness of the framework and the relevance of the identified implications for system design.

**Table 2.** Sustainability dimensions of the SusAF^a^.

Dimension	Description
Social	Concerns relationships between individuals and groups, including trust, communication, participation, inclusion, and the balance of potentially conflicting interests
Individual	Addresses individual well-being, autonomy, dignity, privacy, safety, and the capacity to make informed decisions and exercise agency
Environmental	Focuses on the stewardship of natural resources, including energy consumption, material use, and ecological impacts across the system life cycle
Economic	Encompasses financial and organizational aspects such as value creation, cost structures, governance, and long-term economic viability
Technical	Relates to the longevity and evolution of the system, including maintainability, adaptability, security, scalability, and resilience over time

a SusAF: sustainability awareness framework.

**Table 3.** Orders of sustainability effects in the SusAF^a^.

Order of effect	Description
Immediate effects	Direct impacts from development, deployment, use, and disposal, including technical features and life-cycle energy consumption
Enabling effects	Indirect impacts emerging over time that induce changes in user behavior, clinical practices, or organizational routines
Structural effects	Long-term, systemic changes at the societal level influencing norms, policies, institutions, or health care infrastructures

a SusAF: sustainability awareness framework.

**Figure 5.**
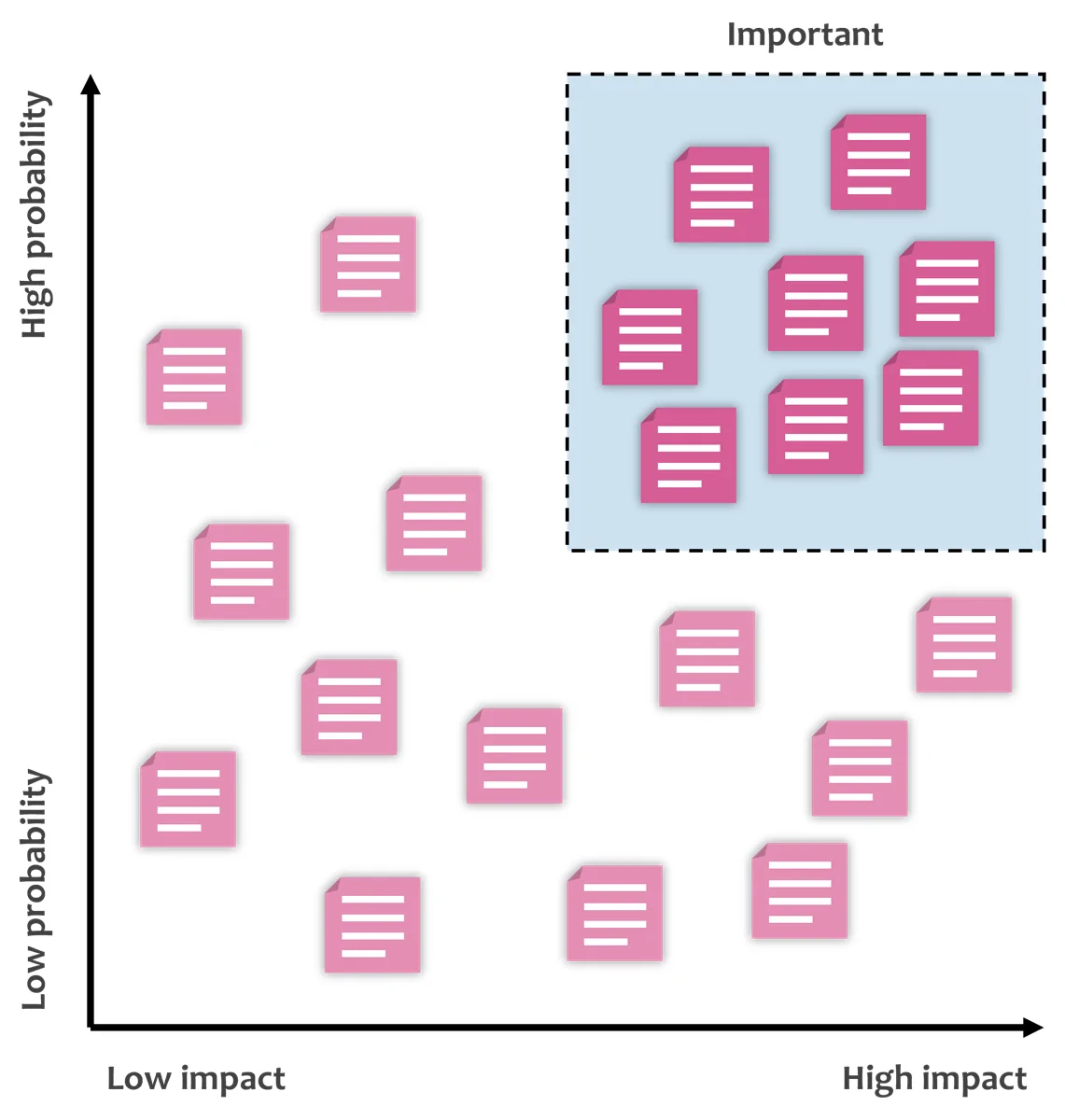
The weighting of impacts within the sustainability awareness framework (SusAF).

**Figure 6.**
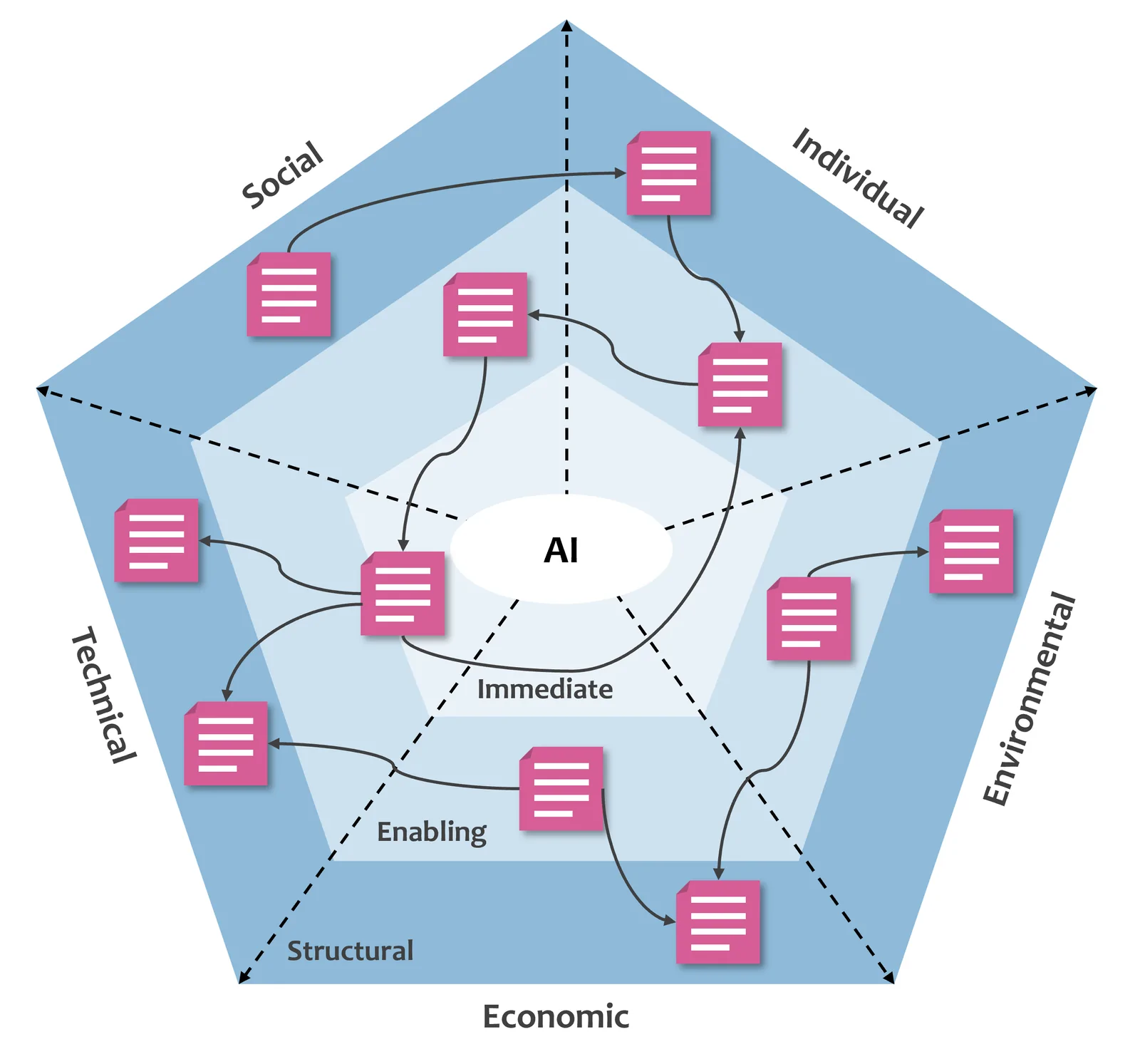
Sustainability awareness diagram (SusAD).

### Workshop Procedure, Data Collection, and Data Analysis

A total of 2 participatory workshops were conducted between July and September 2025, each lasting approximately 4 hours. Workshop 1 focused on stakeholder analysis and the VPC and included 8 participants from clinical and technical domains. Workshop 2 applied the SusAF and involved all 18 stakeholders. Each workshop followed a structured sequence consisting of (1) introduction and contextual framing, (2) guided individual reflection, (3) small-group work in interdisciplinary subgroups, and (4) plenary discussion and consolidation of results. Workshops were moderated by 2 facilitators with backgrounds in socioinformatics and qualitative research, following a predefined facilitation guide to ensure consistency.

The workshops followed a semistructured format combining individual reflection, collaborative exercises, and framework-based activities. Participants worked both individually and in interdisciplinary groups to generate and reflect on design-relevant insights.

Across both workshops, documented materials (structured outputs, field notes, and artifacts) comprised approximately 45 pages of textual data. No audio recordings were made; discussions were documented in real time and consolidated immediately after each session. The use of multiple data sources enabled triangulation and a comprehensive understanding of stakeholder perspectives.

Data were analyzed using an inductive, codebook-supported form of thematic analysis. The analysis followed a staged procedure consisting of (1) familiarization with structured workshop outputs, field notes, and artifacts, (2) open coding of design-relevant needs, risks, tensions, and safeguards, (3) grouping of codes into preliminary categories, (4) comparison of categories across stakeholder analysis, VPC, and SusAF outputs, and (5) iterative refinement into higher-level design requirements.

The stakeholder analysis, VPC, and SusAF frameworks structured data collection and served as sensitizing lenses during interpretation, but they did not predetermine the final themes. Codes were first generated inductively from the documented workshop materials. They were then compared across the 3 frameworks to identify recurring patterns, contradictions, and cross-stakeholder tensions. For example, codes related to “role boundaries,” “medical accountability,” “automation bias,” and “escalation to human professionals” were consolidated into the higher-level requirement of constrained system authority and mandatory human oversight. Similarly, codes concerning “information overload,” “adaptive explanation,” “emotional reassurance,” and “uncertainty communication” informed the requirement of context-sensitive communication.

Coding was conducted independently by 2 researchers, DL and YS, on the complete set of documented materials. Differences in coding and category assignment were discussed until consensus was reached. Categories were retained as higher-level design requirements only when they were supported by multiple data sources or when contested perspectives could be reconstructed as meaningful stakeholder tensions. This procedure enabled triangulation across data sources, including field notes, structured workshop artifacts, and framework-based outputs, as well as across analytical lenses, including stakeholder analysis, VPC, and SusAF.

Workshops were not audio-recorded due to the sensitive clinical context and to foster an open discussion atmosphere. To mitigate potential loss of nuance and selective documentation, field notes were taken by multiple facilitators in parallel, compared after each workshop, and expanded immediately after each session while the discussion context was still fresh. Structured workshop outputs, including written artifacts, prioritization matrices, stakeholder maps, and framework-based templates, were treated as primary qualitative data rather than as supplementary documentation. Field notes were used to contextualize these artifacts, reconstruct stakeholder reasoning, and identify tensions that were not fully captured in the structured templates.

The research team’s dual role as system designers and analysts was addressed through reflexive practices. Researchers documented assumptions and potential biases in analytic memos and revisited them throughout the coding process. Particular attention was given to disconfirming cases and tensions between stakeholder groups. Interpretations were retained only when supported by multiple data sources or when contested perspectives could be reconstructed.

The findings therefore describe stakeholder-informed design and governance requirements rather than evidence of system performance, safety, usability, or clinical effectiveness. Future work should therefore develop and evaluate a prototype, validate the reliability of the RAG-based architecture, and specify the content governance pipeline in detail.

To enhance transparency, illustrative excerpts from field notes and artifacts are reported in the Results section. These are paraphrased for readability and attributed to stakeholder groups to maintain confidentiality.

As the system is in an early conceptual stage, no real-world use data (eg, system logs, user interaction metrics, or adherence data) were collected. Instead, insights are based on structured stakeholder interactions and qualitative analysis.

### Ethical Considerations

This study did not involve direct patient participation or the collection of patient-level health data. The study involved professional stakeholders who contributed expert perspectives in participatory design workshops. All participants were recruited based on their professional expertise and provided informed consent prior to participation.

Formal ethical approval was not required for this expert consultation and qualitative design study in accordance with institutional guidelines on good scientific practice at the Technical University of Munich. Nevertheless, ethical safeguards were applied throughout the research process, including voluntary participation, informed consent, confidentiality, anonymized documentation of workshop outputs, responsible data handling, and anonymized reporting of stakeholder perspectives.

No audio recordings were made. Workshop data were documented through structured artifacts, field notes, and summarized outputs without attributing statements to identifiable individuals. All study materials were stored on access-restricted institutional systems and were accessible only to the research team.

## Results

The following sections present stakeholder-derived design requirements and sociotechnical tensions identified through the participatory co-design process using the VPC and SusAF.

### VPC: Aligning Patient Needs With System Capabilities

The VPC workshop synthesized stakeholder-informed interpretations of the needs of *BRCA1/2* mutation carriers, structured into customer profiles (gains, pains, and customer jobs) and corresponding system responses.

#### The Mutation Carrier Profile: Gains, Pains, and Customer Jobs

Stakeholders described 2 central phases: understanding genetic risk and evaluating preventive options in relation to life plans.

##### Gains

Stakeholders emphasized the need for continuous access to reliable, evidence-based information to support decision-making. Key gains include structured action options, individualized risk information, and preparation for clinical consultations. Emotional needs include empathic communication, continuity of care, and reassurance, as well as multilingual and accessible information.

##### Pains

Fear emerged as a dominant factor, including anxiety related to diagnosis, surveillance, fertility, and family implications. This is compounded by information overload, time pressure, and uncertainty about preventive options. Additional challenges include limited psychosocial support, lack of trustworthy information, language barriers, and financial concerns.

##### Customer Jobs

Mutation carriers aim to regain orientation and control, understand individual risk, and make informed decisions regarding prevention, surveillance, and family planning. Stakeholders highlighted the importance of trust in clinical care and access to understandable information, alongside initial skepticism toward digital tools.

Stakeholders emphasized informational fragmentation during waiting periods, as illustrated by one participant, “patients receive pieces of information from different disciplines, but no coherent picture emerges.” Emotional burden was closely linked to informational uncertainty, with one expert noting that patients fear “making the wrong decision under pressure.” At the same time, clinical stakeholders stressed decision quality and guideline adherence, warning that “simplifying information too much risks misinterpretation.” Stakeholder perspectives differed in how needs were prioritized. Clinical stakeholders emphasized decision quality and guideline alignment, while psychosocial and advocacy stakeholders focused on emotional burden and continuity of support. Technical stakeholders highlighted feasibility, scalability, and interaction design.

### The System’s Value Proposition: Mechanisms for Support

The chat and voice bot was conceptualized as a supportive tool that complements clinical care.

#### Gain Creation

Adaptive communication tailors information to individual needs and supports preparation for clinical consultations, enhancing perceived autonomy.

#### Pain Relievers

The system addresses emotional distress through validation and referral to human support while maintaining clear role boundaries and providing structured, evidence-based information.

#### Trust and Onboarding

Stakeholders emphasized that trust depends on transparent communication, clinical integration, and clear positioning as a non-authoritative informational tool.

#### Information Support

The system provides continuous access to curated information on prevention, fertility, and psychosocial aspects in multilingual and accessible formats.

#### Boundary Management and Governance

The system incorporates safeguards against overreliance, including explicit uncertainty communication, encouragement of clinical verification, and expert-reviewed quality assurance. The system is constrained to an informational and preparatory role and includes default escalation pathways to human professionals.

Across stakeholder groups, three requirements were consistently prioritized: (1) clear communication of system limitations, (2) integration into clinical care pathways, and (3) mechanisms for human oversight and escalation. Other features were considered secondary, indicating that trust-building and governance are foundational design conditions.

### User Case Scenario as an Integrative Result

A narrative user case scenario was developed to represent a mutation carrier’s trajectory from diagnosis to decision-making. It translates abstract requirements into a clinical context, supports alignment on system boundaries (eg, exclusion of longitudinal monitoring and imaging diagnostics), and informs subsequent sustainability and ethical evaluations (see Multimedia Appendix 1).

### Cross-Stakeholder Tensions

The analysis revealed several tensions between stakeholder groups, indicating that system design requires balancing competing priorities rather than addressing isolated needs. The most distinctive contribution of the stakeholder workshops was not the identification of responsible AI principles as such, but the reconstruction of how these principles were negotiated, prioritized, and translated into design requirements in the specific context of *BRCA1/2*-related hereditary cancer care. The 3 core requirements should therefore be understood as outcomes of cross-stakeholder tensions rather than as standalone principles.

A central tension concerned the level of system autonomy. Clinical stakeholders emphasized strict role boundaries and the positioning of the system as a nonauthoritative informational tool, whereas advocacy and psychosocial stakeholders highlighted the need for continuity and emotional support, which may increase reliance on the system.

A second tension involved the depth and complexity of information. Medical professionals favored precise and detailed communication to avoid misinterpretation, while communication-focused stakeholders emphasized simplification and progressive disclosure to prevent cognitive overload.

A third tension emerged between technical scalability and ethical safeguards. Technical stakeholders emphasized efficiency and automation, whereas ethicists and data protection experts prioritized transparency, explainability, and strict governance mechanisms.

These tensions were reflected in contrasting stakeholder statements. For example, clinical stakeholders emphasized strict system boundaries (“the system must not be perceived as giving medical advice”), whereas advocacy-oriented participants stressed the need for continuity (“patients need something that feels like ongoing support, not just information”). Similarly, clinicians highlighted the importance of precision (“details matter to avoid wrong conclusions”), while communication-focused stakeholders emphasized simplification (“too much detail overwhelms patients in this situation”).

These contrasting perspectives indicate that design requirements emerge from negotiated trade-offs rather than uniform stakeholder agreement. These trade-offs informed the subsequent interpretation of onboarding, communication design, quality management, and clinical integration as interdependent governance mechanisms rather than isolated design features.

### Results From the SusAF

Stakeholders identified sustainability-related impacts across all 5 SusAF dimensions. Rather than treating these dimensions as separate domains, the analysis showed that sustainability in this context depends on whether the system can support *BRCA1/2* mutation carriers during a clinically and emotionally sensitive waiting period without displacing human care or amplifying misinterpretation.

### Context-Specific Social and Individual Implications

Across the social and individual dimensions, stakeholders emphasized that 24-hour availability, multilingual access, and the possibility to ask sensitive questions in a low-threshold format could support orientation, self-efficacy, and preparation for clinical consultations. These benefits were considered particularly relevant for topics such as hereditary risk, fertility, preventive surgery, family communication, and psychosocial burden. At the same time, participants warned that decontextualized genetic information could increase anxiety, distort risk perception, or contribute to stigmatization and discrimination. The main design implication was therefore not simply to increase access to information but to provide adaptive, progressively disclosed, and clearly bounded information with transparent escalation pathways to human professionals.

### Technical, Data-Related, and Governance Implications

The technical dimension was closely linked to these psychosocial and clinical concerns. Stakeholders viewed a curated knowledge base and RAG as promising mechanisms for source-based information provision, but only if combined with expert review, continuous content maintenance, robust data protection, and explicit communication of uncertainty. Automation bias was identified as a key risk, particularly because users may interpret chatbot responses as medical authority in moments of uncertainty or distress. Technical sustainability therefore depends on maintaining a clear distinction between informational preparation and clinical decision-making.

### Environmental and Economic Conditions

Environmental and economic effects were discussed as conditional rather than inherent benefits of digital support. Remote access may reduce unnecessary travel or preparatory consultations, and structured information may help clinical encounters become more focused. However, these potential benefits depend on energy-efficient infrastructure, long-term maintenance capacity, sustainable financing, and regulatory compliance. Stakeholders therefore emphasized that digitalization should not be assumed to be sustainable by default; its value depends on governance, clinical integration, and continued institutional responsibility.

The SusAF analysis showed that the most relevant sustainability concerns in this use case are not generic efficiency gains but the interaction between access, psychological safety, genetic data sensitivity, clinical accountability, and long-term governance. Without clear boundaries, transparency, and integration into clinical workflows, features intended to empower users may also amplify risks such as misinterpretation, overreliance, exclusion, or inequity, underlining the dual-use character of conversational AI in health care contexts.

### Stakeholder Reflections on the Process

The postworkshop evaluation (n=14/18) indicates a broadly positive reception of the SusAF process. Ten respondents rated its usefulness as high to very high, and none reported low relevance. Eight participants considered the knowledge gained to justify the time investment, while 6 participants assessed the effort-benefit ratio as moderate.

Participation influenced perspectives, with 8 respondents reporting moderate to strong changes in their understanding of medical chatbot requirements. These shifts reflected increased awareness of heterogeneous user needs and structural constraints of clinical practice, including limited time and the need for interdisciplinary coordination. Interdisciplinary exchange was identified as a key success factor, with 13 participants rating it as highly helpful.

Regarding implementation, stakeholders emphasized that the system should function as a supportive, preparatory tool rather than a standalone solution. Key requirements include transparent communication of system limitations, robust informed consent, and clear positioning of medical professionals as primary points of contact. Participants also highlighted the importance of cultural sensitivity through multilingual and accessible communication.

## Discussion

The findings highlight how responsible conversational AI in hereditary cancer care depends on constraining system authority, embedding human oversight, and integrating systems into clinical care pathways.

### Onboarding Process and Informed Consent

The stakeholders understood onboarding as a central sociotechnical mechanism for shaping trust and role perception within the care pathway. In the context of hereditary cancer, initial digital interactions appear closely linked to processes of trust formation and emotional safety. A human-mediated onboarding process emerges as particularly important for positioning the chat and voice bot as a supportive tool rather than a replacement for clinical expertise. Introducing the system through medical professionals may therefore contribute to reduced anxiety and clearer role expectations within the care pathway.

From an ethical perspective, informed consent can be understood as a multilayered and ongoing communicative process rather than a one-time procedural step. This includes explicitly communicating system limitations, potential inaccuracies, and the need for human verification. Such transparency is critical for mitigating automation bias and safeguarding individual autonomy, while also reducing cognitive overload and reinforcing clinical responsibility as part of sustainable system use.

The findings further indicated that onboarding should be conceptualized as adaptive and revisitable over time, particularly as patients transition between different phases of decision-making. As individuals move from initial risk assessment to surgical considerations, informational needs and emotional states evolve, requiring corresponding adjustments in how consent and system use are framed.

### Linguistic Abilities and Communication Strategies

The findings indicated that linguistic configuration represented a central component of the sociotechnical integration of the chat and voice bot. It could be conceptualized along 3 interrelated dimensions, including sociolinguistic awareness, inclusivity, and strategic communication. The ability to balance an appropriate medical register with adaptive language complexity emerged as particularly critical. This includes adjusting terminology and depth of explanation to individual patient backgrounds, ranging from barrier-free communication for less experienced users to more technical discourse when appropriate. Such adaptability appeared essential for fostering resonance and preventing alienation.

Linguistic inclusivity further extended beyond translation toward a multilingual and multimodal communication architecture. Enabling interaction in a user’s preferred language may strengthen accessibility and perceived conversational intimacy. At the same time, bridging the digital–physical divide through multimodal outputs, such as combined voice, text, and structured materials for clinical preparation, appeared to support continuity of care and integration into clinical workflows.

With regard to communication strategies, the findings pointed to the importance of balancing factual clarity with empathic responsiveness. While concise and action-oriented responses are necessary to avoid ambiguity and prevent misinterpretation, acknowledging emotional states remains central to user-centered interaction. In this context, epistemic transparency—including explicit communication of system limitations—emerged as a key mechanism for aligning professional credibility with responsible use.

Taken together, these observations suggest that effective communication design requires the integration of sociolinguistic sensitivity and factual precision. This integration enables the system to address users as individuals rather than as abstract clinical cases, thereby supporting both informational and emotional needs.

Finally, communication must be understood as embedded within broader sociocultural contexts. This includes sensitivity to topics such as fertility, body image, and genetic responsibility, as well as to variations in health literacy, perceptions of medical authority, and attitudes toward preventive interventions. Cultural competence, therefore, extends beyond multilingual support and represents a core condition for responsible system design.

### Usability and User Experience

In the workshops, usability and user experience (UX) extended beyond interface design and constituted central conditions shaping the perceived clinical fit of the system. In the context of hereditary cancer risk, UX can be understood as a form of emotional scaffolding, particularly for users experiencing cognitive and emotional overload.

The SusAF analysis indicates that perceived autonomy is closely linked to interaction design. Features such as progressive disclosure and modular content enable users to regulate the depth and pace of information, thereby supporting agency and reducing the risk of disempowerment. In this sense, usability also appears to function as a proxy for institutional trust, as transparent interaction design and explicit cues for escalation to human professionals reinforce confidence in the system’s appropriate role.

At the same time, usability must be responsive to heterogeneous user groups. This includes ensuring that voice-based interaction and accessibility features reduce barriers without oversimplifying medically complex information. Technical robustness and error transparency are equally critical, as usability-related failures—such as latency or unmasked hallucinations—carry ethical implications by potentially fostering misplaced trust.

### Quality Management

Across the participatory workshops, stakeholders emphasized that quality management for medical conversational agents should be embedded within existing structures of clinical governance rather than treated as a purely technical concern. The chat and voice bot can thus be understood as a preparatory and informational tool whose legitimacy depends on clearly defined institutional responsibilities and explicit role boundaries between automated support and human medical expertise.

Accordingly, quality assurance appears to rely on clinically curated knowledge bases, expert validation processes, and established medical standards, rather than being delegated to the system itself. In this context, quality management emerges as a continuous and iterative process requiring ongoing expert review and system refinement.

With regard to accountability, responsibility was consistently located within the clinical organization rather than attributed to the system. Quality management therefore functions as a mechanism for ensuring traceability, transparency, and professional accountability, supported by clear escalation pathways to human professionals.

This governance perspective also clarifies the added value of a purpose-built system compared with general-purpose LLMs. While general-purpose systems may provide fluent and accessible responses, their use alone does not establish clinical accountability, validated content governance, institutionally defined role boundaries, or reliable escalation pathways. For *BRCA1/2* mutation carriers, these governance conditions were not peripheral safeguards but central design requirements, because users may otherwise mistake conversational fluency for medical authority in moments of uncertainty or distress, as illustrated by the risk-framing and triage example outlined above.

### Environmental and Economic Trade-Offs of Digital Support

Environmental and economic considerations emerged as secondary but relevant governance concerns. In contrast to the social, individual, technical, and legal issues, these effects were less specific to hereditary cancer care, but they remain important for assessing whether conversational AI can be responsibly sustained in clinical practice.

From an environmental perspective, potential benefits arise primarily through the reduction of physical travel, as remote access to specialized information may decrease unnecessary in-person consultations and associated emissions. However, these benefits are highly dependent on system design. Energy-intensive processes, including model training, inference, and supporting infrastructure, may offset such gains if not carefully managed. Environmental outcomes thus emerge as indirect consequences of architectural and governance decisions, requiring energy-efficient infrastructures and minimized computational overhead.

Similarly, economic implications reflect a tension between potential efficiency gains and structural costs. While shifting preparatory informational tasks to the system may relieve clinical staff and enable more focused patient encounters, the development, certification, maintenance, and regulatory compliance of medical-grade AI systems represent substantial and ongoing financial commitments.

Taken together, sustainability cannot be assumed as an automatic outcome of automation. Instead, both environmental and economic viability depend on governance conditions, including transparent financing structures, institutional responsibility for long-term maintenance, and alignment with health care reimbursement systems.

### Data Protection, Privacy, and Legal Requirements

The findings indicated that legal and regulatory compliance is not merely a boundary condition but a central determinant of trust, system acceptance, and responsible integration into clinical care. Data protection requirements, in particular, can be understood as core design conditions that shape system architecture, communication strategies, and governance structures.

The deployment of the chat and voice bot falls within the scope of the General Data Protection Regulation (GDPR; EU) 2016/679, as it involves the processing of personal data (Article 4(1) GDPR; cf. Court of Justice of the European Union [CJEU], Endemol Shine, C-740/22), including chat logs and genetic risk information. The institution determining the purposes and means of processing acts as the controller (Article 4(7) GDPR).

From a design perspective, data minimization and user control emerge as central requirements. An anonymization-oriented approach (Recital 26(5) GDPR) may be implemented through pseudonymized access codes. Where datasets cannot be linked to identifiable individuals by any party, the information is considered anonymized and thus falls outside the scope of the GDPR (cf. CJEU, Single Resolution Board [SRB], C-413/23 P).

Compliance with core principles such as lawfulness, fairness, and transparency (Article 5(1)(a) GDPR) must be operationalized in ways that remain accessible to users. While scientific research may benefit from specific provisions (Article 89 GDPR), deployment in clinical practice requires a valid legal basis, such as a medical contract (Article 6(1)(b) GDPR) or consent ([46], para 48). For special categories of personal data, including genetic and broadly defined health data (cf. CJEU, C-21/23), explicit consent (Article 9(2)(a) GDPR) or Article 9(2)(h) GDPR in conjunction with Section 22 of the German Bundesdatenschutzgesetz (Federal Data Protection Act; BDSG) applies, provided appropriate safeguards are ensured.

At the same time, the findings highlight that extensive legal information may itself contribute to cognitive overload, particularly in emotionally vulnerable situations. This suggests the need for layered privacy communication, combining concise summaries with access to more detailed information, in line with transparency requirements under Article 13 and 14 GDPR [47].

Concerns regarding automated decision-making further underline the importance of clearly defined system boundaries. Although the system was designed to provide informational support without producing legally binding or directly consequential decisions, maintaining this distinction is critical to avoid regulatory risks (Article 22 GDPR; see also CJEU, Schufa, C-634/21) and unintended overreliance.

Beyond data protection, stakeholders pointed to the relevance of the EU AI Act (Regulation [EU] 2024/1689), particularly regarding transparency obligations (Article 50(1)). Clear disclosure of AI interaction was therefore considered essential to support informed use and prevent misinterpretation.

The classification of the system under the Medical Device Regulation (MDR; Regulation [EU] 2017/745) remains context-dependent [48-50]. Maintaining the system’s role as an informational and preparatory tool, rather than engaging in monitoring or treatment (Article 2(1) MDR) [51,52] appears critical for avoiding more stringent regulatory requirements. Current legislative developments (the European Commission’s proposed COM [2025] 1023 final) [53] may affect the interaction between the MDR and the AI Act.

At the national level, the German Genetic Diagnosis Act (GenDG) requires explicit consent for processing genetic data (Section 11 GenDG). These requirements are closely linked to broader questions of liability and professional responsibility [54]. Rather than attributing responsibility to the system, accountability remains anchored in clinical institutions. In this context, technical safeguards, such as constrained system prompts and conservative response strategies, function as mechanisms to reinforce the system’s supportive role and limit potential liability (Sections 280, 823 German Civil Code–BGB).

Overall, legal and regulatory requirements emerged as integral components of system design. Rather than being addressed retrospectively, they shape core design decisions, including system boundaries, communication strategies, and the positioning of human professionals as primary decision makers.

Compared with the informal use of general-purpose LLMs, an institutionally governed system would also allow clearer allocation of controller responsibilities, data minimization strategies, consent procedures, and accountability structures within the clinical setting.

### Summary: Reframing Responsibility in Conversational AI for Health Care

Taken together, this sociotechnical analysis suggests that responsibility in conversational AI is not an intrinsic system property but a relational and organizationally situated outcome. Rather than arising from technical accuracy or compliance alone, it emerges from concrete design decisions, including limiting system authority, embedding escalation pathways, and ensuring transparency about uncertainty.

These findings point to a shift in emphasis from optimizing autonomous systems toward designing sociotechnical arrangements that preserve relational care and professional responsibility. More broadly, they indicate that responsibility in medical AI is less a function of model performance than of how system capabilities are deliberately positioned, limited, and governed within clinical contexts.

This study contributes stakeholder-informed insights into how principles of responsible AI can be translated into context-sensitive design considerations for clinical implementation. Rather than proposing new normative frameworks, its central contribution lies in showing how responsible AI requirements are shaped through practical negotiations between competing clinical, psychosocial, technical, and organizational priorities, including accuracy versus accessibility, emotional support versus role boundaries, and scalability versus governance.

Although situated in hereditary cancer care, the identified tensions—including those between autonomy and safety, accessibility and accuracy, and scalability and governance—are likely to extend to other domains of AI-supported, preference-sensitive decision-making in health care.

### Limitations

The qualitative and exploratory design prioritizes analytical transferability over statistical generalizability. While this enables in-depth, context-sensitive insights, the findings may not fully represent all clinical settings.

The purposive sampling of stakeholders strengthens practical relevance but introduces potential selection bias. The sample predominantly reflects expert perspectives from the European, specifically German, health care context. Validation in different cultural and regulatory environments is therefore required.

Construct validity may be affected by varying interpretations of abstract concepts such as sustainability. This was addressed through the use of established frameworks (VPC and SusAF) and structured conceptual framing. Nevertheless, reactive bias remains a limitation, as participants in workshop settings may exhibit socially desirable or consensus-oriented responses. Mitigation strategies included neutral facilitation and anonymous postworkshop surveys.

Because workshops were not audio-recorded, the analysis may have missed nuances of interaction, phrasing, or disagreement that would have been available in verbatim transcripts. This limitation was mitigated through parallel field note-taking by multiple facilitators, immediate postworkshop consolidation, triangulation with structured artifacts and framework-based outputs, and consensus-based interpretation by 2 researchers.

Differences in digital literacy among participants likely influenced perceptions of AI-related risks. Although a common baseline was established through introductory explanations, such variation cannot be fully controlled in interdisciplinary settings. In addition, the interpretive subjectivity inherent in qualitative analysis may affect results, despite collaborative coding and iterative validation processes.

The absence of direct patient involvement is a substantive methodological limitation, particularly because the envisioned conversational AI system is intended to support *BRCA1/2* mutation carriers. Patient perspectives were represented indirectly through psycho-oncological and advocacy expertise, which provided clinically and experientially informed approximations of user needs but cannot substitute for direct accounts of lived experience. Future research should therefore involve mutation carriers directly under appropriate ethical and clinical safeguards to validate, refine, and potentially challenge the stakeholder-derived requirements.

The research team’s dual role as system designers and analysts may have influenced problem framing and interpretation. While this enabled contextual sensitivity and iterative refinement, it also required continuous reflexive practices to mitigate potential confirmation bias.

The findings are context-dependent and may not be directly transferable to settings lacking institutional oversight, digital literacy, or clearly defined professional responsibility structures.

Although the manuscript discusses key German/EU regulatory considerations, a definitive legal classification of the system under the MDR and the EU AI Act was beyond the scope of this exploratory design study and should be examined in future work together with legal and clinical implementation experts.

Overall, the findings should be understood as context-sensitive rather than definitive, highlighting the need for longitudinal research and broader population-based studies to assess the long-term deployment of responsible AI in health care.

### Summary and Conclusion

This study provides a structured account of how established responsible AI principles are negotiated and translated into stakeholder-derived design requirements for conversational AI in *BRCA1/2*-related hereditary cancer care. The findings suggest that effective implementation depends less on increasing system autonomy than on constraining and embedding AI within clinical governance structures.

Across stakeholder groups, responsible use was consistently associated with transparent communication of system limitations, clearly defined role boundaries, and mechanisms for human oversight. Against the backdrop of widely accessible general-purpose LLMs, these findings suggest that the added value of a purpose-built system lies not in conversational access alone, but in clinically bounded governance, validated content, institutional accountability, data protection, and reliable escalation pathways. These elements emerge as central conditions for trust, appropriate use, and the prevention of over-reliance.

By combining stakeholder analysis, the VPC, and the SusAF, this study demonstrates how abstract principles of responsible AI can be translated into context-sensitive design considerations. Importantly, these considerations arise from negotiated tensions rather than from uniform stakeholder agreement.

Future research should directly involve *BRCA1/2* mutation carriers, evaluate real-world use, and examine how such systems interact with clinical workflows over time. In addition, the findings underline that systematic alignment with evolving regulatory frameworks should not be treated as a downstream compliance task but as an integral part of responsible design, particularly in relation to GDPR, MDR, GenDG, the EU AI Act, and institutional accountability in the German/EU health care context.

## Supplementary material

10.2196/97869 Multimedia Appendix 1 One pager/user case scenario, “Knowledge against fear,” providing support for *BRCA1/2* carriers.

## Abbreviations

BDSG: Bundesdatenschutzgesetz (Federal Data Protection Act)
BGB: Bürgerliches Gesetzbuch (German Civil Code)
CJEU: Court of Justice of the European Union
COREQ: Consolidated Criteria for Reporting Qualitative Research
EU: European Union
FBREK: German Consortium for Familial Breast and Ovarian Cancer
FORCE: Facing Our Risk of Cancer Empowered
GDPR: General Data Protection Regulation
GenDG: German Genetic Diagnosis Act
IPDAS: International Patient Decision Aid Standards
LLM: large language model
MDR: Medical Device Regulation
RAG: retrieval-augmented generation
SRB: Single Resolution Board
SusAD: sustainability awareness diagram
SusAF: sustainability awareness framework
UX: user experience
VPC: value proposition canvas
VSD: value-sensitive design
WHO: World Health Organization
xAI: explainable AI
