# Supplementary material for: Conversational AI in Hereditary Cancer Care: Sociotechnical Study of Responsible Design Requirements
Source: JMIR Hum Factors. 2026 Sep 30;13:e97869. doi: 10.2196/97869 (PMC13626551; doi:10.2196/97869)
Supplement: Supplementary file 1 [file humanfactors-v13-e97869-s001.docx]

**‘Knowledge against fear’ – support for BRCA1/2 carriers**

*One-pager in preparation for the stakeholder workshop*

**Barbara, a BRCA1 mutation carrier caught between concern, education and decision-making**

Barbara is 34 years old, a teacher, in a stable relationship – and recently learned about her BRCA1 germline mutation. After her mother developed BRCA1-associated ovarian cancer, Barbara underwent predictive genetic testing: there was a 50% chance that her mother had passed on this disease-causing mutation to her. She has now had an appointment at the tumour genetics clinic. The figures are clear: **her lifetime risk of breast cancer is approximately 72%, and her risk of ovarian cancer is approximately 44%.**

Since she found out about the implant, she has been torn. On the one hand, she is grateful to have found out early. On the other hand, her thoughts constantly revolve around questions such as: **Should I have surgery as a precaution? What consequences would this have for my body and my fertility? What does all this mean for my relationship?**

At the gynaecological clinic, she experiences real relief for the first time. Gynaecology, human genetics, radiology and psycho-oncology work hand in hand here. The gynaecologist from the team spends 45 minutes with her during an initial consultation, talking openly about prevention options and side effects, breast reconstruction and hormone replacement therapy in the context of surgical menopause. Barbara no longer feels like a ‘case’, but like a woman with individual needs. But the time is quickly over, and the next patient is already waiting outside the door. Approximately one in 400 to 500 women in the general population share Barbara's fate of having such a mutation. The probability is significantly higher for index patients with a family history of the disease – up to 30% or more, depending on the family constellation.

Until her second appointment with the gynaecologist, which is not for another six weeks, she has time to gather more information and prepare specific questions: What exactly happens during preventive surgery? How likely am I to remain cancer-free? Are there any alternatives? But Barbara realises how difficult it all is. The information on the internet is contradictory and difficult to understand. Complicated terms such as ‘mastectomy’, “oophorectomy” and ‘chemopreventive’ are swirling around in her head. She wants a clear, reliable overview, preferably with specific and medically sound options for action. At the same time, she is burdened by vague fears: of the menopause, of a suspicious finding in her breast, of the decision itself. She also feels tension in her relationship. The topic of family planning suddenly takes on a new light. What if I decide too late? What if I decide too quickly? The concern about passing on the mutation to her children hangs unspoken in the air. Barbara feels alone. She says:

‘I need a decision that I can stand behind – with the feeling that it suits me!’

**Chat and Voice Bot (CVB): Scientifically based guidance during a sensitive phase**

Barbara has six weeks until her second appointment with the tumour genetics doctor – and many questions. During this sensitive phase, she uses CVB that provides her with scientifically sound information. CVB is by no means intended to replace medical advice, but rather to help her better understand information, clarify her own values and needs, find her own point of view, and prepare specifically for the upcoming second consultation.

CVB explains to Barbara, for example, how a BRCA1 mutation affects cancer risk, what preventive options are available, and what questions she could ask during her second consultation. CVB adapts the language and depth of the content individually depending on her prior medical knowledge. Barbara can also ask questions on topics such as fertility, quality of life, psychological support, or health insurance. If necessary, CVB refers her directly to suitable specialists and organisations, such as the BRCA Network, a patient organisation with self-help groups throughout Germany, including in Barbara's home town of Munich.

**The CVB thus helps Barbara to gain more confidence and orientation at her own pace through sound knowledge.**
